# Advanced Glycation End Products (AGEs) in Diet and Skin in Relation to Stool Microbiota: The Rotterdam Study

**DOI:** 10.3390/nu15112567

**Published:** 2023-05-30

**Authors:** Jinluan Chen, Djawad Radjabzadeh, Carolina Medina-Gomez, Trudy Voortman, Joyce B. J. van Meurs, M. Arfan Ikram, André G. Uitterlinden, Robert Kraaij, M. Carola Zillikens

**Affiliations:** 1 Department of Internal Medicine, Erasmus MC, University Medical Center Rotterdam, Doctor Molewaterplein 40, 3015 GD Rotterdam, The Netherlands; j.chen@erasmusmc.nl (J.C.);; 2 Department of Epidemiology, Erasmus MC, University Medical Center Rotterdam, 3015 GD Rotterdam, The Netherlands

**Keywords:** advanced glycation end products, microbiota, diet, skin, stool, gut

## Abstract

Background: Advanced glycation end products (AGEs) are involved in age-related diseases, but the interaction of gut microbiota with dietary AGEs (dAGEs) and tissue AGEs in the population is unknown. Objective: Our objective was to investigate the association of dietary and tissue AGEs with gut microbiota in the population-based Rotterdam Study, using skin AGEs as a marker for tissue accumulation and stool microbiota as a surrogate for gut microbiota. Design: Dietary intake of three AGEs (dAGEs), namely carboxymethyl-lysine (CML), *N*-(5-hydro-5-methyl-4-imidazolon-2-yl)-ornithine (MGH1), and carboxyethyl-lysine (CEL), was quantified at baseline from food frequency questionnaires. Following up after a median of 5.7 years, skin AGEs were measured using skin autofluorescence (SAF), and stool microbiota samples were sequenced (16S rRNA) to measure microbial composition (including alpha-diversity, beta-dissimilarity, and taxonomic abundances) as well as predict microbial metabolic pathways. Associations of both dAGEs and SAF with microbial measures were investigated using multiple linear regression models in 1052 and 718 participants, respectively. Results: dAGEs and SAF were not associated with either the alpha-diversity or beta-dissimilarity of the stool microbiota. After multiple-testing correction, dAGEs were not associated with any of the 188 genera tested, but were nominally inversely associated with the abundance of *Barnesiella*, *Colidextribacter*, *Oscillospiraceae UCG-005*, and *Terrisporobacter*, in addition to being positively associated with *Coprococcus*, *Dorea*, and *Blautia*. A higher abundance of *Lactobacillus* was associated with a higher SAF, along with several nominally significantly associated genera. dAGEs and SAF were nominally associated with several microbial pathways, but none were statistically significant after multiple-testing correction. Conclusions: Our findings did not solidify a link between habitual dAGEs, skin AGEs, and overall stool microbiota composition. Nominally significant associations with several genera and functional pathways suggested a potential interaction between gut microbiota and AGE metabolism, but validation is required. Future studies are warranted, to investigate whether gut microbiota modifies the potential impact of dAGEs on health.

## 1. Introduction

Advanced glycation end products (AGEs) are a group of molecules formed nonenzymatically, after the initial attachment of reducing sugars to amino groups of proteins, lipids, or nucleic acids [1]. Long-lived tissues are more prone to AGEs accumulation, and sustain damage due to the formation of cross-links, the modification of proteins, or from inflammation due to their binding to the receptor for AGEs (RAGE) [2,3,4]. Evidences are gathering for their possible involvement in aging [5] and age-related diseases, such as cardiovascular diseases, dementia, and diabetes [6].

AGEs are also generated from food processing, especially under high temperatures and low moisture [7]. Dietary AGEs (dAGEs) were associated with elevated inflammation, as well as incidences of type 2 diabetes [8] and pancreatic cancer [9], but these associations are still inconclusive, and their underlying mechanisms remain unclear. Aside from entering the circulation, it was estimated that a large fraction (20–50%) of dAGEs are excreted in feces [10]. Thus, an interaction with the commensal microbiota in the intestinal tract is likely. Gut microbiota composition and metabolic activities can vary in response to dietary factors, which in turn may influence host health through their involvement in inflammation, metabolic processes, immunoregulation, etc. [11,12].

Exposure to dAGEs was reported to increase colon permeability in rats [13]. Their cross-linking structures can also hinder microbe mobility [14]. dAGEs may activate downstream inflammatory responses once recognized by RAGE, which is highly expressed in the intestinal tract [15]. They may also influence microbial and host metabolism, such as by reducing the abundance of microbes that produce health-provoking short-chain fatty acids (SCFAs) and induce insulin resistance [16]. In contrast, some dAGEs were substrates for SCFA production [17,18]. Potential interactions of dAGEs and the gut microbiota have been summarized in Supplementary Figure S1. An intervention study of one month’s dAGE restriction was reported to already alter the gut microbiota of patients undergoing peritoneal dialysis, as measured in stool [19]. However, the way in which AGEs interact with the human gut microbiota is still largely unexplored, and data from large population studies are lacking [20,21]. Gut microbial metabolism and fermentation in turn provide a large panel of metabolites to the host, including AGEs and their precursors [22]. Gut microbiota may also indirectly influence AGE levels by contributing to systemic inflammation and oxidative stress.

To sum up, whether dietary AGEs modulate the gut microbiota or whether activities of gut microbiota influence the AGE burden in vivo may be relevant for host health but remains largely unknown. To explore the intricate connections between dietary AGEs, gut microbiota, and host AGEs accumulation, we obtained data from the population-based Rotterdam Study on stool microbiota as a surrogate for gut microbiota, estimating dAGEs from dietary information, and using skin AGEs as a reflection of AGE accumulation in long-lived tissues [23]. We examined the associations of dietary and skin AGEs with the overall diversity, dissimilarity, and the taxonomic and functional abundance of the stool microbiota.

## 2. Subjects and Methods

### 2.1. Study Population

The Rotterdam Study is a general population-based cohort consisting of three subcohorts of inhabitants from the Rotterdam suburb Ommoord who were invited to participate. All the participants of our study were from the third subcohort of the Rotterdam Study (RS III), which started in 2006, and the majority of whom were of European ancestry. In total, N = 3932 participants (≥45 years old) were included at baseline (RS III 1st visit) with follow-up examinations every 4–6 years. The design and objectives of the Rotterdam Study have been extensively described previously [24].

The dietary intakes of three types of AGEs, namely carboxymethyl-lysine (CML), *N*-(5-hydro-5-methyl-4-imidazolon-2-yl)-ornithine (MGH1), and carboxyethyl-lysine (CEL), were estimated based on a food frequency questionnaire (FFQ) at baseline (RS III 1st visit) for 2676 participants. Skin AGEs were measured (*n* = 1167), and stool samples for microbiota analyses were collected (*n* = 1420) during RS III 2nd visit (the median follow-up time being 5.7 years later).

A total of 1120 participants had data taken on both dAGEs and stool microbiome, after excluding 47 participants with implausible energy intake (<500 kcal/d or >5000 kcal/d). Of this 1120, a further 27 participants were excluded because the stool samples had been in ambient temperature for more than 6 days, or for an unknown duration. Another 37 participants were excluded either because they had used antibiotics less than 1 month before stool sample collection, or because their antibiotic usage was unknown. An additional 3 participants (*n* = 3) with remaining outlying values in dietary AGEs (>±6 SD) were additionally excluded (Details in Supplementary Figure S2). For the stool microbiota and SAF analysis, 752 participants had both measurements available. From this group, we excluded 2 participants with outlying values of SAF (>±4 SD), and a further 32 participants based on the samples’ time in ambient temperature and their antibiotic usage, leaving 718 participants for analysis (Supplementary Figure S3).

### 2.2. Estimation of Dietary AGEs and Other Dietary Characteristics

When visiting the RS research center, participants received a 389-item food frequency questionnaire (FFQ). This FFQ collected detailed information, including food types, frequencies, portions, and some preparation methods of 389 food and beverage items covering the preceding month. The FFQ has been validated to properly rank subjects for nutrient intake in other studies among Dutch adults [25,26].

Briefly, dAGEs were calculated from the FFQ using reference contents of three types of AGEs (CML, MGH1, and CEL) in 190 food items from a Dutch database [27], as well as from another reference database on CML contents in 257 food items from Northern Ireland [28]. Both databases reported the content of protein-bound AGEs, determined using ultraperformance liquid chromatography-tandem mass-spectrometry (UPLC-MS/MS). Details of the method were described elsewhere [29]. We used energy-adjusted AGE intake for our analyses in order to reduce reporting bias related to measurement error [30]. Dietary energy intake was calculated using the Dutch Food Composition Table (NEVO). Overall diet quality was approximated in a diet quality score (with a range of 0–14), reflecting adherence to the Dutch Dietary Guidelines [31].

### 2.3. Measurement of Skin AGEs as SAF

Skin AGEs were measured as SAF, using AGE Reader™ (DiagnOptics B.V., Groningen, The Netherlands) on the inner skin of the dominant forearm at the Rotterdam Study research center during the RS III 2nd visit. This assessed the overall level of AGEs in the skin, based on the fluorescent property of some AGEs and their correlations with other AGEs. Details of the measurement have been described elsewhere [32].

### 2.4. Stool Microbiota Profiling and Processing

Fecal samples were collected at home by the participants and sent to Erasmus MC by mail. Upon arrival, samples were recorded and stored at −20 °C. DNA was isolated in accordance with the manufacturer’s protocol (Arrow Stool DNA; Isogen Life Science, Utrecht, The Netherlands). The V3 and V4 variable regions of the 16S rRNA gene were amplified using the 309F-806R primer pair and sequenced on an Illumina MiSeq sequencer (MiSeq Reagent Kit v3, 2 × 300 bp) with an average depth of 50,000 reads per sample [33].

Raw reads from the MiSeq were demultiplexed using a custom script to separate sample FASTQ files based on the dual index. Primers, barcodes, and heterogeneity spacers were trimmed off using tagcleaner v0.16 [34]. Trimmed FASTQ files were loaded into R v4.0.0 with the DADA2 [35] package (version 1.18.0). Quality filtering was performed in DADA2 using the following criteria: trim = 0, maxEE = c(2,2), truncQ = 2, rm.phix = TRUE. Filtered reads were run through the DADA2 Amplicon Sequence Variant (ASV) assignment tool to denoise, cluster, and merge the reads. ASVs were assigned a taxonomy from the SILVA version 138.1 rRNA database [36] using the RDP naïve Bayesian classifier [37]. The resulting data tables were combined into a phyloseq object using phyloseq R package (version 1.38.0) [38] and a phylogenetic tree was generated using the phangorn R package (version 2.6.2) [39] based on the sequences of the ASVs, and then added to the phyloseq object.

ASVs with a read count of less than 0.05% of the total reads, as well as those observed in less than 1% of the total samples, were precluded from the analysis. Technical covariates included the season of stool production, number of total reads, time in the mail (assessing the days of the samples’ being in an ambient temperature), and batch (marking each run of DNA isolation and DNA sequencing).

### 2.5. Assessment of Covariates and Population Characteristics

Covariates were assessed at the RS III 2nd visit. Information on smoking, alcohol intake, physical activity, medications used during the past week, and medical history was collected during home interviews. Smoking status was categorized as never, past, or current smoker, based on cigarette, cigar, and pipe smoking information. Alcohol intake was assessed in glasses/day and harmonized to grams of alcohol per day. The use of antibiotics was provided by the participants when stool samples were collected and included no use of antibiotics, used in less than one month, 1–3 months, or 3 months–1 year before sample collection. Height and weight were measured at the research center and BMI (kg/m^2^) was calculated as weight divided by height squared. Fasting blood samples were obtained, and HDL cholesterol, total cholesterol, triglycerides, creatinine, and glucose were measured using routine techniques. LDL cholesterol (mmol/L) was calculated with the Friedewald formula. The estimated glomerular filtration rate (eGFR) was calculated using the CKD-EPI equation. Diabetes was defined as a fasting glucose of ≥7.0 mmol/L, or a nonfasting glucose of ≥11.0 mmol/L and/or the use of glucose-lowering medications, and was verified with medical records.

### 2.6. Statistical Analysis

All statistical analyses were performed in R Studio (Version 1.4.1717, R Version 4.1.0). The number of independent tests was calculated based on the correlation matrix between outcomes of interest using the method described by Li and Ji [40]. Bonferroni correction was used to maintain a low false positive rate in the presence of multiple testing. Results which had a *p*-value less than 0.05 but did not pass the *p*-value threshold after correction were regarded as nominally significant.

#### 2.6.1. Characteristics of Microbial Composition

Three indexes were used to describe the overall alpha diversity of the stool microbiota, namely the Shannon Index, the Inverse Simpson Index, and the number of ASVs observed. The calculation of the former two indexes was carried out using the diversity function in vegan (R package) based on the abundance of all ASVs after initial quality control and filtering. Centered log-ratio (CLR) transformation of the abundance of the individual taxon was performed using the transform function in the Microbiome R package. Zeros in taxon abundance were replaced with a pseudo count of min (nonzero abundance)/2 before taking logs. Beta dissimilarity, which measures the between-participants dissimilarities of stool microbiota composition, was calculated as Aitchison distance, i.e., Euclidean distance, based on the CLR transformed abundance of all ASVs, using the vegdist function from the vegan package [41].

#### 2.6.2. Microbial Pathway Prediction

We used the PICRUSt2 (v.2.5.0) tool to obtain predicted microbial functions based on the MetaCyc dataset and the ASV table [42,43]. In short, the prediction was performed using the default EPA-NG polygenetic replacement option and MinPath biological pathway reconstruction, based on Enzyme Commission (EC) numbers. ASVs with less than 0.05% of the total read count, or which were present in less than 1% of the samples, were precluded.

#### 2.6.3. dAGEs and Microbiota Analyses

Descriptives of the lifestyle, clinical, dietary, and stool microbiota characteristics of the participants are presented in tertile groups of low, medium, and high CML intake. Results on CML were presented in the main text since it has been extensively studied in the literature, and the estimation in diet was more accurate based on reference values from more food items than it was for the other two types of AGEs.

(1) dAGEs and alpha diversity

Associations between dAGEs and alpha diversity indexes were analyzed in multiple linear regression models, adjusting for factors that would potentially influence the level of AGEs in the diet or gut microbiota composition. Two linear regression models were constructed where the Shannon Index, Inverse Simpson Index, or the number of observed ASVs were the outcomes, respectively: model 1 was adjusted for age, sex, seasons of stool production, number of total reads, time in the mail, and batches of DNA isolation and sequencing. Model 2 was further adjusted for covariates that were reported to influence stool microbiota substantially or were associated with dAGEs, including the use of PPI and antibiotics, alcohol consumption, BMI, diabetes, diet quality score, energy intake, and smoking status.

(2) dAGEs and beta dissimilarity

To visualize sample distances, we extracted the first two principal components (PC) that explained the most variation of stool microbiota composition based on CLR transformed ASV abundance. The samples were plotted in dAGE tertile groups with a two-dimensional ordination plot based on the first two PCs. We tested for differences in microbiota beta dissimilarity among dAGEs tertile groups using permutational multivariate analysis of variance (PERMANOVA) with 999 times permutations using the adonis2 function in vegan package. All covariates and categorical dAGEs were entered into PERMANOVA models sequentially.

(3) dAGEs and abundance of individual genera

After aggregating all the ASVs to the genus level, the analysis was confined to 188 genera. We studied the association using linear regression, accounting for confounders in models described previously. To achieve a better model fit and reduce the chance of false positive discoveries due to the compositional nature of the microbiome data, the dependent variable (i.e., abundance of each genus) was CLR transformed. Heteroscedasticity and model diagnostics were inspected by plotting the linear regression residuals against the predicted outcome. All results in this study were derived from model 2 for genera presented in at least 30% of all participants, unless specified otherwise.

(4) dAGEs and predicted microbial pathways

For further insight into the link between dAGEs and microbial metabolic potential, we studied the association between dAGEs and the putative microbial metabolic pathways in the linear regression models. adjusting for the covariates described previously. The abundances of predicted metabolic pathways were CLR transformed and used as the outcome in our models (360 pathways were analyzed).

#### 2.6.4. Stool Microbiota and SAF

Sex-specific, age-adjusted SAF was obtained as the residuals of SAF regressed against age in women and men, separately. Participants were grouped into tertiles of sex-specific, age-adjusted SAF to obtain descriptive statistics, which then underwent beta dissimilarity testing after adjusting for other covariates. We examined the relationship between stool microbiota and SAF through the same serial analyses as those of the dAGEs study, but now with stool microbiota traits as exposures and SAF as the outcome (except for analysis on beta dissimilarity by SAF groups). Confounders were adjusted in two models: Model 1 was adjusted for age, sex, seasons of stool production, number of total reads, time in the mail, and DNA isolation and sequencing batches; Model 2 was additionally adjusted for the use of PPI and antibiotics, alcohol consumption, BMI, diabetes, eGFR, and smoking status. For all analyses, model 2 was considered to be the main model.

#### 2.6.5. Sensitivity Analysis

We repeated the dAGEs analyses after further excluding dAGEs outside of the mean ± 4 SD range (*n* = 14) so as to exclude results driven by them, although the potential impact from dietary components is more likely to be observed with exceptional intake. We also repeated all the analyses after further excluding participants who had used antibiotics within 3 months prior to stool sample collection (*n* = 67). Only the overlapping results for genera presented in at least 30% of the population were discussed further, as we are more certain that these results were more robust to influences from outlying dAGE values, antibiotics, and the sparse presence of microbes in the population.

#### 2.6.6. Imputation of Missing Values

Missing values in the use of antibiotics, smoking, alcohol, eGFR, and BMI were imputed through multiple imputation (m = 10) in R (MICE package, version 3.14.0).

## 3. Results

### 3.1. dAGEs and Stool Microbiota

#### 3.1.1. Descriptive statistics

For the dAGEs and microbiome study (*n* = 1052), clinical and lifestyle characteristics of the total study population are shown in Table 1. The mean (SD) age of the participants was 62.5 (5.5) years, with 59% being female. Mean (SD) daily intake was 2.5 (0.9) mg for CML, 29.5 (8.1) mg for MGH1, and 2.5 (0.9) mg for CEL after energy adjustment. Diabetes mellitus was present in 107 (10%) participants (mostly type 2). Shannon Index, Inverse Simpson Index, and the number of observed ASVs were higher in the low CML group compared to the medium and high CML groups.

#### 3.1.2. dAGEs and Overall Diversity and Dissimilarity of the Stool Microbiota

dAGEs were not associated with all three measures of microbial alpha diversity (see Supplementary Table S1). In PCA analysis, the first two PCs accounted for 4.5% and 2.5% of the total variance of the microbiota composition, respectively. We did not observe an obvious separation of individual microbiota for the dAGE tertile groups in the ordination plot based on the first two PCs (Supplementary Figure S4). No significant difference was observed in beta dissimilarity among dAGEs tertile groups in PERMANOVA (Supplementary Table S2). The results persisted after restricting the analysis to participants (*n* = 971) who had dAGEs within the mean ±4 SD range and had not used antibiotics for at least three months prior to sample collection.

#### 3.1.3. dAGEs and Microbial Abundance

**Figure 1:**
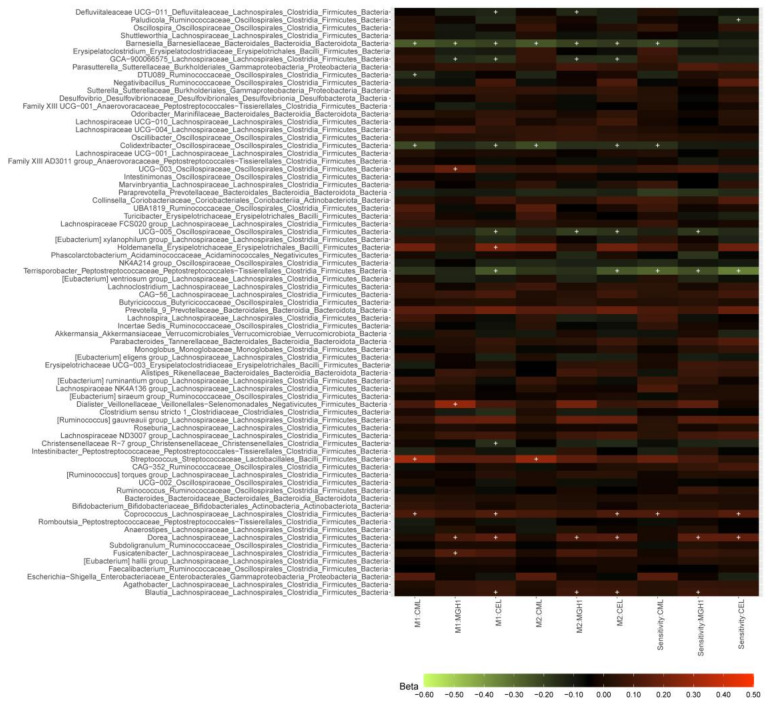
dAGEs-microbial taxa associations in the total population and after further excluding individuals in sensitivity analyses. M1, M2: results from model 1 and model 2 in the total study population (N = 1052). Sensitivity: sensitivity analysis results from model 2 after further excluding participants who had used antibiotics in less than three months before stool sample collection and whose dAGEs intake exceeding the mean ± 4 SD range (N = 971). Associations between dAGEs and the abundance of 188 genera were derived from linear regression model 2 in 1052 and 973 participants, respectively. The color of the boxes represents the direction of the association, with red being positive and green being negative and color lightness implies the magnitude of beta coefficients obtained from linear regression analyses. Beta coefficients are adjusted differences of the centered log-ratio transformed genera abundance associated with one SD difference of dAGEs. “+” denotes *p* < 0.05. The associations were adjusted for age, sex, season of stool production, number of total reads, batches of DNA isolation and sequencing, and time in the mail in model 1, and in model 2 further for the use of PPI, the use of antibiotics, diabetes, BMI, diet quality score, energy intake, alcohol intake, and smoking status. Source data in Supplementary Tables S3–S5.

The three dAGEs showed 31 nominally significant associations (*p* < 0.05) with 188 genera, but no association passed the statistical significance threshold after Bonferroni correction (*p* < 0.0002 with 300 independent tests) (Supplementary Tables S3 and S4). Further exclusion of participants in sensitivity analyses did not change the results substantially (Supplementary Table S5). The associations of dAGEs with genera that were present in ≥30% of participants are summarized in Figure 1. Of them, a higher dAGE intake was associated with a lower abundance of genus *Barnesiella* of the Barnesiellaceae family, *Colidextribacter* and *UCG-005* of the Oscillospiraceae family, and *Terrisporobacter* of the Peptostreptococcaceae family (and *GCA_900066575* of the Lachnospiraceae family with a *p* < 0.1 in the sensitivity analysis), and a higher abundance of genera *Coprococcus*, *Dorea*, and *Blautia* of the Lachnospiraceae family, in both the main and sensitivity analyses.

#### 3.1.4. dAGEs and Microbial Pathways

We tested the associations of three types of dAGEs with 360 predicted microbial metabolic pathways. A total of 49 nominally significant associations were observed (*p* < 0.05) but none of them reached statistical significance (*p* < 0.0003) after Bonferroni correction (number of independent tests: 148). Among the nominally significantly associated pathways that presented in at least 30% of the samples, inversely associated pathways included the superpathway of *N*-acetylneuraminate degradation, pyruvate fermentation to acetone, acetyl-CoA fermentation to butanoate II, the superpathway of UDP-*N*-acetylglucosamine-derived O-antigen building block biosynthesis, biotin biosynthesis II, L-glutamate degradation V (via hydroxyglutarate), adenosylcobalamin biosynthesis I (early cobalt insertion), the superpathway of polyamine biosynthesis II, and L-lysine fermentation to acetate and butanoate. Positively associated pathways included inosine-5′-phosphate biosynthesis III, L-lysine biosynthesis II, the superpathway of geranylgeranyldiphosphate biosynthesis I (via mevalonate), peptidoglycan biosynthesis II (staphylococci), mevalonate pathway I, and the superpathway of 2,3-butanediol biosynthesis. (Details in Supplementary Table S6).

### 3.2. Stool Microbiota and SAF

Characteristics of all the participants included in the SAF study and arranged in sex-specific, age-adjusted SAF tertile groups are shown in Supplementary Table S7. The low SAF group showed slightly higher alpha diversity (Supplementary Table S8), but no association was observed in multiple linear regression models (Supplementary Tables S9 and S10). In the PCA analysis, the first two PCs accounted for 4.6% and 2.5% of the total variance of the microbiota, respectively. We also did not observe an apparent separation of the SAF tertile groups based on the first two PCs (Supplementary Figure S5). No significant difference in Aitchison distance was observed among sex-specific, age-adjusted SAF tertiles in PERMANOVA after adjusting for covariates in model 2 (Details in Supplementary Table S11).

Regarding individual taxa, a higher abundance of genus *Lactobacillus* from the Lactobacillaceae family was associated with a higher SAF after Bonferroni correction (*p*-value cut-off: *p* < 0.0007) but microbes of this genus only presented in a small fraction (23%) of samples (Supplementary Table S10). Among genera that presented in more than 30% of the studied population, SAF was inversely associated with the abundance of genera *[Eubacterium] ventriosum* group, *Anaerostipes*, *Roseburia*, *Lachnospiraceae NK4A136* group, *[Eubacterium] eligens* group, and *Lachnospiraceae UCG-001* from the Lachnospiraceae family, *Oscillospiraceae UCG-005* from the Oscillospiraceae family, and genus *Sutterella* from the Sutterellaceae family. SAF was nominally positively associated with the abundance of the genus *Negativibacillus* from the Ruminococcaceae family.

We observed three pathways, present in at least 30% of the participants, that were nominally (*p* < 0.05) associated with SAF: positive associations with the superpathway of L-aspartate and L-asparagine biosynthesis, pyrimidine deoxyribonucleosides salvage, and L-histidine degradation I. However, none of them were statistically significant after Bonferroni correction (*p* < 0.0007, based on 74 independent tests) (Supplementary Table S12).

## 4. Discussion

We investigated dietary and skin AGEs in relation to gut microbiota composition in a general population cohort using stool microbiota as a surrogate. dAGEs were not associated with microbial alpha diversity or beta dissimilarity. We observed nominally significant associations between dAGEs and genera abundance, but none of them survived multiple testing correction. Despite a higher alpha diversity being observed in the lowest SAF tertile, neither alpha diversity nor beta dissimilarity was associated with SAF, but the abundance of the genus *Lactobacillus* was positively associated with SAF.

Limited studies were available on dAGEs and microbiota concerning intervention in humans and animals and in vitro fermentation [13,16,19,44,45,46,47,48], and no general population-based data were available. Glycated products were often suggested to reduce the alpha diversity and abundance of SCFA producing microbes in stool microbiota [49], but some studies reported elevated SCFA production and indicated potential health benefits [46,50,51]. One mice study showed a high dAGEs diet led to inflammation and altered gut microbiota composition and this was reversed following low dAGEs intake [52]. However, our study did not show any apparent dissimilarity of stool microbiota in the dAGE groups, paralleling a 4-week dAGEs intervention study in obese individuals that did not observe apparent different microbial composition [53]. This could be explained by the fact that our study population may be rather homogenous in dietary AGEs, while the stool microbiota varies drastically with factors such as age, diet, and comorbidities. Further, we studied the habitual diet that the gut microbiota might have adapted to [54].

For the analysis of dAGEs and genera abundance, with a sample size of 1052, we only have 50.8% power to demonstrate the largest observed difference (beta = 0.28, alpha = 0.003), suggesting our study is underpowered to detect any smaller differences after Bonferroni correction. Despite that, a higher level of dAGEs was nominally associated with a lower abundance of *Barnesiella*, *Colidextribacter*, *Oscillospiraceae UCG-005*, *Terrisporobacter*, and *Lachnospiraceae GCA_900066575*, and a higher abundance of *Coprococcus*, *Dorea*, and *Blautia*. The differentially abundant genera found in our study differ from those found in other studies, which could be partially attributed to differences in study duration, amount of AGEs, possible human-specific adaptations of the microbiota to thermally processed food [55], and absence of interaction with host physiology in in vitro studies.

Disbiome, an online dataset documenting published associations between microbes and diseases, gives hints of potential health relevance [56]. A reduced *Barnesiella* was seen in chronic kidney disease, autism, inflammatory bowel disease, and obesity, among others. A reduced *Terrisporobacter* abundance was seen in obesity, autism, and type 2 diabetes. *Lachnospiraceae GCA_900066575* was inversely associated with BMI [57]. A higher *Dorea* abundance was reported in atrial fibrillation, autism, irritable bowel syndrome, and non-alcoholic fatty liver disease. A higher *Blautia* was seen in atrial fibrillation, major depressive disorder, and type 1 diabetes. A higher abundance of *Coprococcus* was also associated with diets rich in sugars but also linked to health-promoting traits, such as the maintenance of microbial homeostasis. In addition, *Coprococcus* and *Blautia* are potential SCFA producers [58,59]. It is worth noting that both higher and lower abundances of some genera were seen in multiple diseases, suggesting that the patterns of disturbance in the entire microbiota might be more informative than variations of a single genus.

We also examined dAGEs in association with microbial pathways, which are thought to be more relevant to certain health risks than compositional variation alone, such as type 1 diabetes in infants [60]. Our results point to the hypothesis that exposing the gut microbiota to a higher dAGE intake may (1) result in lower SCFAs, which are substrates for the positively associated pathways, such as the “superpathway of 2,3-butanediol biosynthesis”, as well as products for the inversely associated pathways such as “*N*-acetylneuraminate degradation”, “acetyl-CoA fermentation to butanoate II”, and “L-lysine fermentation to acetate and butanoate”; (2) modulate cholesterol levels through the mevalonate pathway I; and (3) modulate the redox status by influencing the pathways that affect the NAD+/NADH ratio and L-glutamate. However, further research is necessary as none of these associations remained statistically significant after multiple testing correction.

As for skin AGEs, both alpha diversity and beta dissimilarity were not associated with SAF. It is worth noting that some covariates also act as intermediates in the association (e.g., diabetes), which could have led to overadjustment. We did not observe a consistent pattern of associations for phylogenetically related taxa; thus, it seems unlikely that tissue AGEs originate from a group of related microbes. This is supported by the lack of association between microbial functions and SAF, although we also lack the statistical power to detect these associations. These results imply that overall gut microbial diversity may not be a major contributor to long-term AGE accumulation in distant tissues, such as the skin.

On the genus level, the abundance of *Lactobacillus* (present in 23% of participants) was associated with a higher SAF. Although *Lactobacillus* is present in fermented foods and is considered a probiotic, a higher abundance in stool microbiota was also seen in obesity and type 2 diabetes, two conditions that increase AGEs. Among all the nominally significant associations observed, a lower abundance of potential SCFA producers [61], *Eubacterium ventriosum* group, *Anaerostipes*, *Roseburia*, *Lachnospiraceae NK4A136* group, *Lachnospiraceae UCG-001*, and *Oscillospiraceae UCG-005*, was nominally associated with a higher SAF. Reduced abundance of the inversely associated genera in the stool microbiota was also observed in multiple diseases, such as: autism, colorectal cancer, Crohn’s disease for *Eubacterium ventriosum* group; inflammatory bowel disease and diabetes for *Anaerostipes*; chronic kidney disease, hypertension, Parkinson’s disease for *Roseburia*; dementia and obesity for *Lachnospiraceae NK4A136*; and diabetes and primary biliary cholangitis for *Sutterella*. The abundance of the associated genera could further be influenced by diet. For instance, *[Eubacterium] eligens* group, which was nominally inversely associated with SAF, was also inversely associated with the intake of AGE-forming fructose [62]. *Negativibacillus*, which was nominally positively associated with SAF, was also associated with ultra-processed foods [63]. Our findings imply that gut microbes may influence SCFAs levels and host health, or participate in the metabolism of AGE-related compounds in the diet and further affect tissue AGEs.

The strengths of this study include a relatively large sample size and a deeply phenotyped population that allows for the control of many potential confounders. We also focused on three representative protein-bound AGEs from the habitual diet. They are more likely to enter the gut microbiota habitat than free AGEs. The dAGEs estimation was based on a detailed 389-item FFQ. Additionally, SAF can reflect tissue AGE accumulation over the long term.

Limitations included that our findings need to be replicated, to reduce the possibility of chance findings. Moreover, our results should be interpreted in light of limited power, imperfect quantification of dAGEs, and potential residual confounding, especially from the diet. The results were restricted to the elderly Dutch population. We also acknowledge limitations inherent to microbiota analysis, including that linear regression might not be the best method to analyze compositional microbiome data.

To gain further insight into the interaction of AGEs and gut microbiota, larger sample sizes are needed to increase the statistical power. Dietary intervention studies and longitudinal studies combined with microbial functional and metabolic data are also warranted if we are to understand the causality of any associations observed. Studies in earlier life stages are needed to understand the health relevance across the lifespan. The link of AGEs with intestinal pathophysiology should also be elucidated, as a recent mice study revealed a critical role of CML in brain aging, and a microbiota-dependent increase in intestinal permeability was key to this process [64]. Studies may also explore the role of the gut microbiome in modifying the relationship between dAGEs and health outcomes. For instance, some microbes such as *Collinsella intestinalis* can degrade dAGEs to innocuous products [65]. This may help explain the inconsistent observations on dAGEs and health risks.

In conclusion, our human population study showed only nominally significant associations between dAGEs and stool microbiota, unlike intervention studies in animals. However, this does not mean that reducing dAGEs will not be beneficial as we assessed the cross-sectional associations and prospective intervention studies are needed to confirm the observations. A higher abundance of *Lactobacillus* was associated with a higher skin AGEs. We await other independent studies to verify this finding. Further research is also required to investigate the causality and health relevance of the associations we and others observed between AGEs and the stool microbiota.

## Figures and Tables

**Table 1:** Characteristics of all participants included for dAGEs and stool microbiome analyses, presented in tertile groups of CML intake.

Characteristic	Total	Tertile Groups of Dietary CML Intake		
		Low CML	Medium CML	High CML
N	1052	351	350	351
Age, y	62.5 ± 5.5	63.0 ± 5.4	62.6 ± 5.6	62.0 ± 5.4
Sex (female)	623 (59)	187 (53)	206 (59)	230 (66)
Smoking status				
Never smoker	348 (33)	99 (28)	125 (36)	124 (36)
Ex-smoker	536 (51)	187 (53)	171 (49)	178 (51)
Current smoker	163 (16)	64 (18)	52 (15)	47 (13)
Physical activity	50.8 (22.1, 88.4)	46.5 (21.2, 82.8)	52.5 (22.5, 87.3)	52.9 (23.6, 92.8)
Alcohol consumption, gram/day	8.6 (1.6, 8.6)	8.6 (3.7, 15.0)	8.6 (1.6, 8.6)	6.4 (1.6, 8.6)
BMI, kg/m^2^	27.3 ± 4.5	27.2 ± 4.3	27.2 ± 4.2	27.5 ± 4.9
Diabetes	107 (10)	36 (10)	32 (9)	39 (11)
Use of PPI, *n* (%)	140 (13)	53 (15)	42 (12)	45 (13)
Antibiotic usage				
No (*n*, %)	861 (82)	281 (80)	289 (83)	291 (83)
Within 1 m prior to collection (*n*, %)	0 (0)	0 (0)	0 (0)	0 (0)
1 m–3 m prior to collection (*n*, %)	67 (6)	25 (7)	21 (6)	21 (6)
3 m–1 y prior to collection (*n*, %)	124 (12)	45 (13)	40 (11)	39 (11)
Diet quality score	7 (6, 8)	7 (6, 8)	7 (6, 8)	7 (6, 9)
Energy intake, kCal/day	2235 (1860, 2718)	2247 (1855, 2765)	2152 (1776, 2599)	2302 (1953, 2752)
Protein intake, g/day	89 ± 26	85 ± 26	86 ± 24	96 ± 26
Fat, g/day	79 (61, 101)	77 (58, 98)	77 (59, 96)	82 (67, 105)
Carbohydrate intake, g/day	261 ± 86	262 ± 90.50	251 ± 87	270 ± 81
CML intake, mg/day	2.5 ± 0.9	1.64 ± 0.46	2.40 ± 0.19	3.49 ± 0.76
MGH1 intake, mg/day	29.5 ± 8.1	24.85 ± 6.09	29.64 ± 5.67	33.93 ± 9.24
CEL intake, mg/day	2.50 ± 0.9	1.98 ± 0.64	2.50 ± 0.61	3.01 ± 0.95
Microbial diversity				
Shannon Index	3.99 ± 0.44	4.02 ± 0.40	3.98 ± 0.42	3.97 ± 0.49
Inverse Simpson Index	32.4 ± 14.55	33.1 ± 14.1	31.5 ± 14.4	32.6 ± 15.1
Number of observed ASVs	159 ± 56.5	161 ± 60	158 ± 56	157 ± 54
Time in mail	1 (1, 2)	1 (1, 2)	1 (1, 2)	1 (1, 2)
Season of sample production				
Spring (*n*, %)	261 (25)	77 (22)	87 (25)	97 (28)
Summer (*n*, %)	189 (18)	67 (19)	57 (16)	65 (19)
Autumn (*n*, %)	319 (30)	100 (28)	114 (33)	105 (30)
Winter (*n*, %)	283 (27)	107 (30)	92 (26)	84 (24)
Number of reads	27,201 (18,243, 34,394)	28,442 (18,737, 34,609)	26,276 (17,673, 34,990)	26,910 (19,291, 33,312)

dAGEs, dietary advanced glycation end products; BMI, body mass index; PPI, proton pump inhibitors; CML, carboxymethyl-lysine; MGH1, *N*-(5-hydro-5-methyl-4-imidazolon-2-yl)-ornithine; CEL, carboxyethyl-lysine. Values are shown for nonimputed data. Values are counts (valid percentages), means ± standard deviations, or median (interquartile range) in case of a skewed distribution.

## Data Availability

The data presented in this study are available on request from the management team of the Rotterdam Study (secretariat.epi@erasmusmc.nl). The data are not publicly available due to restrictions based on privacy regulations and informed consent of the participants.

## Supplementary Materials

The following supporting information can be downloaded at: https://www.mdpi.com/article/10.3390/nu15112567/s1, Figure S1: Summary of existing literature information on the mechanisms; Figure S2: Population inclusion and exclusion flow diagram for dAGEs and stool microbiome analyses; Figure S3: Population inclusion and exclusion flow diagram for stool microbiome and SAF analyses; Figure S4: Ordination plot of microbiota beta dissimilarity among dAGE tertile groups; Figure S5: Ordination plot of microbiota beta dissimilarity among SAF tertile groups; Table S1: Associations between dietary AGEs intake and measures of alpha diversity in the total population; Table S2: Summary of PERMANOVA of beta dissimilarity by dAGEs tertile groups; Tables S3–S5: dAGEs-taxa associations in the total population; Table S6: dAGEs-pathway associations in the total population; Table S7: Characteristics of participants included for stool microbiome and skin autofluorescence analyses and by tertile groups of skin autofluorescence; Table S8: The association between measures of alpha diversity and SAF; Tables S9 and S10: The associations between abundance of taxonomical units and SAF in the total population; Table S11: Summary of PERMANOVA of beta dissimilarity by SAF tertile groups; Table S12: The associations between microbial metabolic (MetaCyc) pathways and SAF in the total population.
