# Supplementary material for: Advanced Glycation End Products (AGEs) in Diet and Skin in Relation to Stool Microbiota: The Rotterdam Study
Source: Nutrients. 2023 May 30;15(11):2567. doi: 10.3390/nu15112567 (PMC10255453; doi:10.3390/nu15112567)
Supplement: Supplementary file 1 [file nutrients-15-02567-s001.zip › Supplementary tables and figures_AGEs and microbiome_20230331.pdf]

Figure S1 Summary of existing literature information on the mechanisms

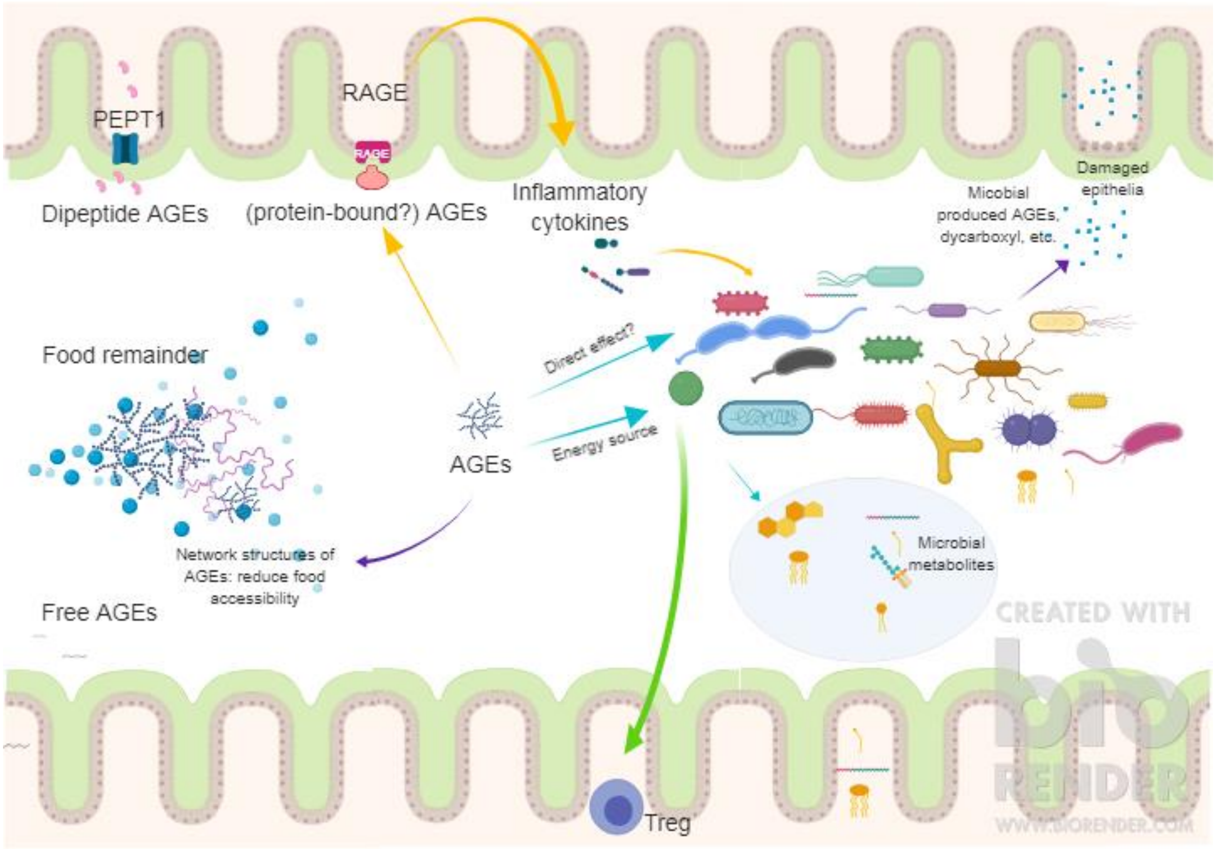

Figure S2 Population inclusion and exclusion flow diagram for dAGEs and stool microbiome analyses.

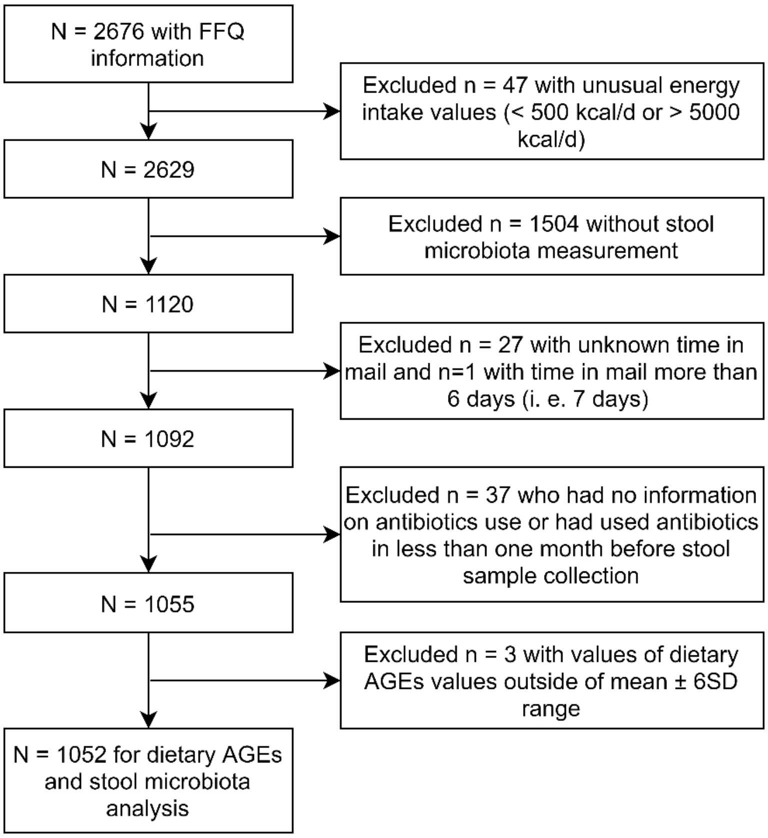

dAGEs, dietary advanced glycation end products; FFQ, food frequency questionnaire; SD, standard deviation.

Figure S3 Population inclusion and exclusion flow diagram for stool microbiome and SAF analyses.

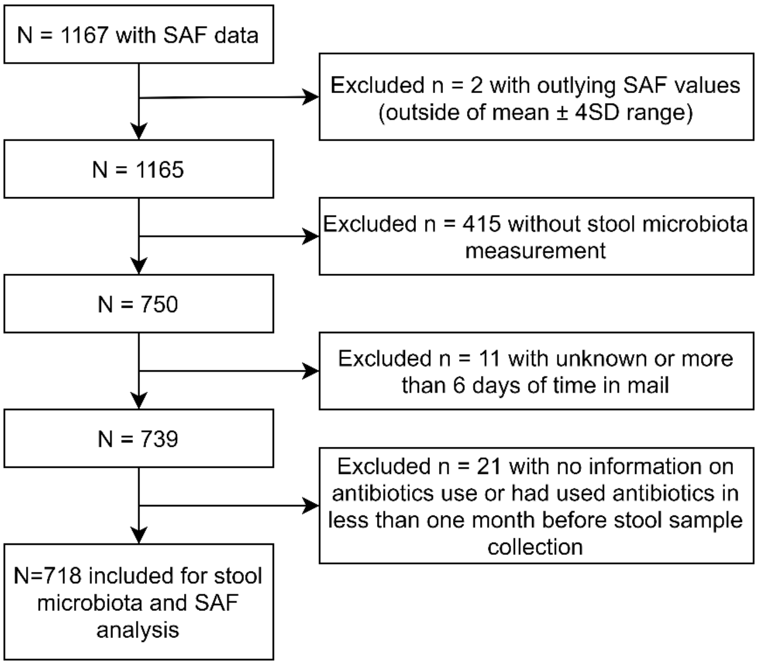

SAF, skin autofluorescence; SD, standard deviation.

Figure S4 Ordination plot of microbiota beta dissimilarity among dAGE tertile groups

(A)

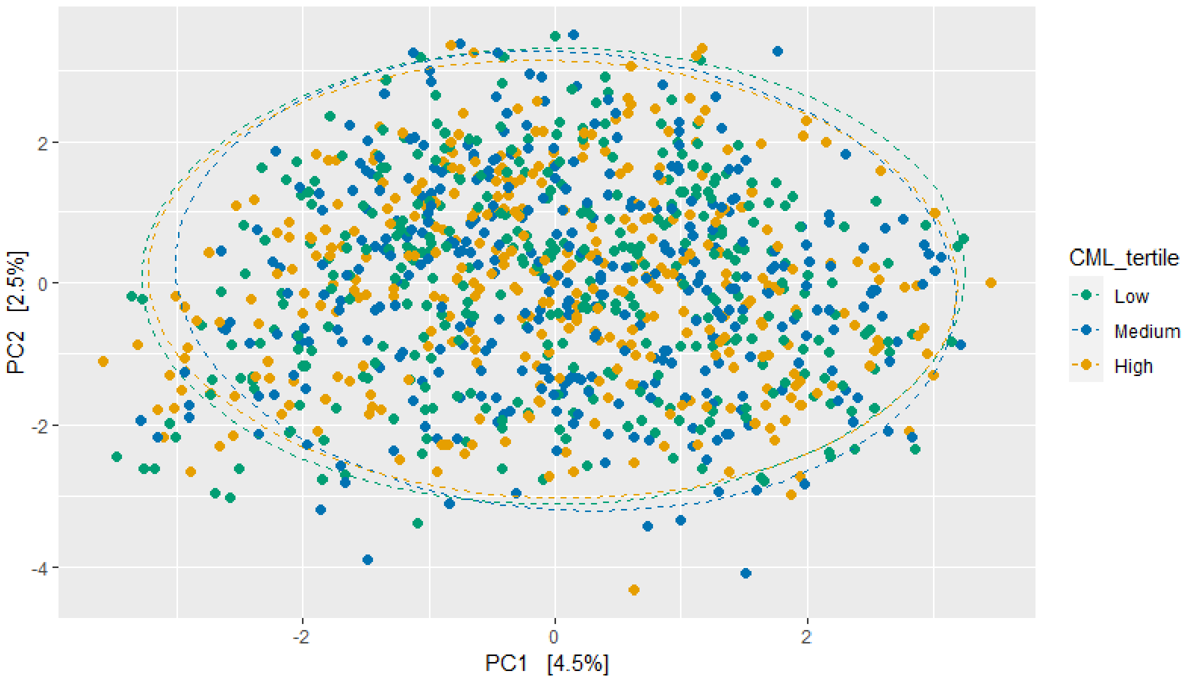

(B)

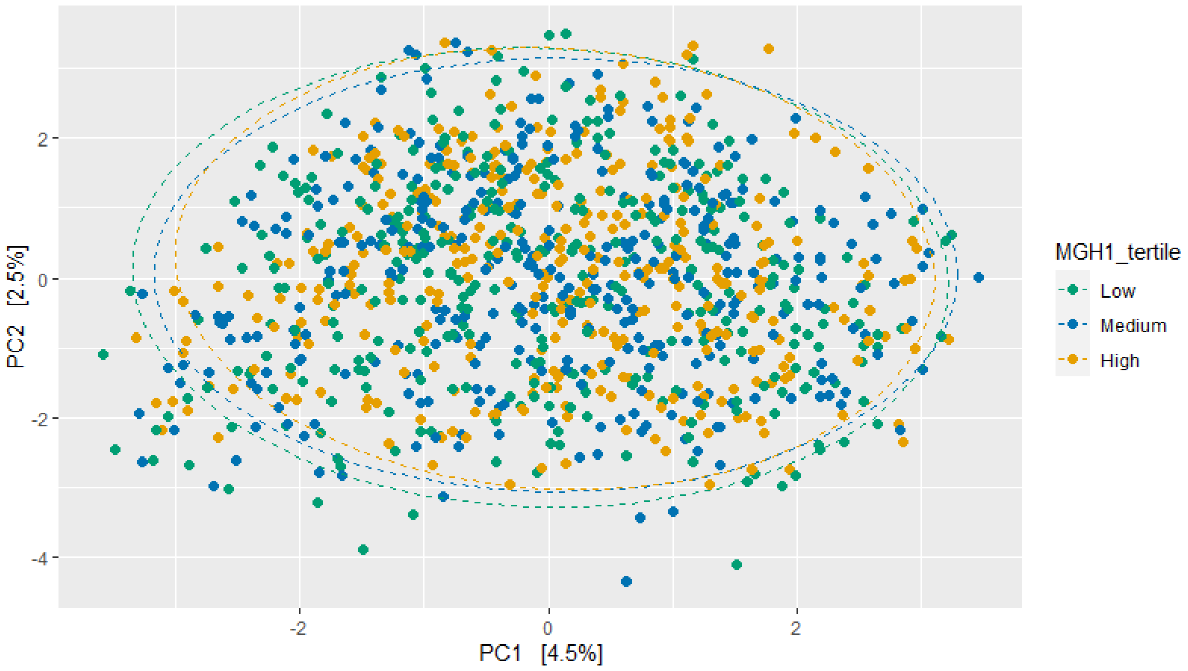

(C)

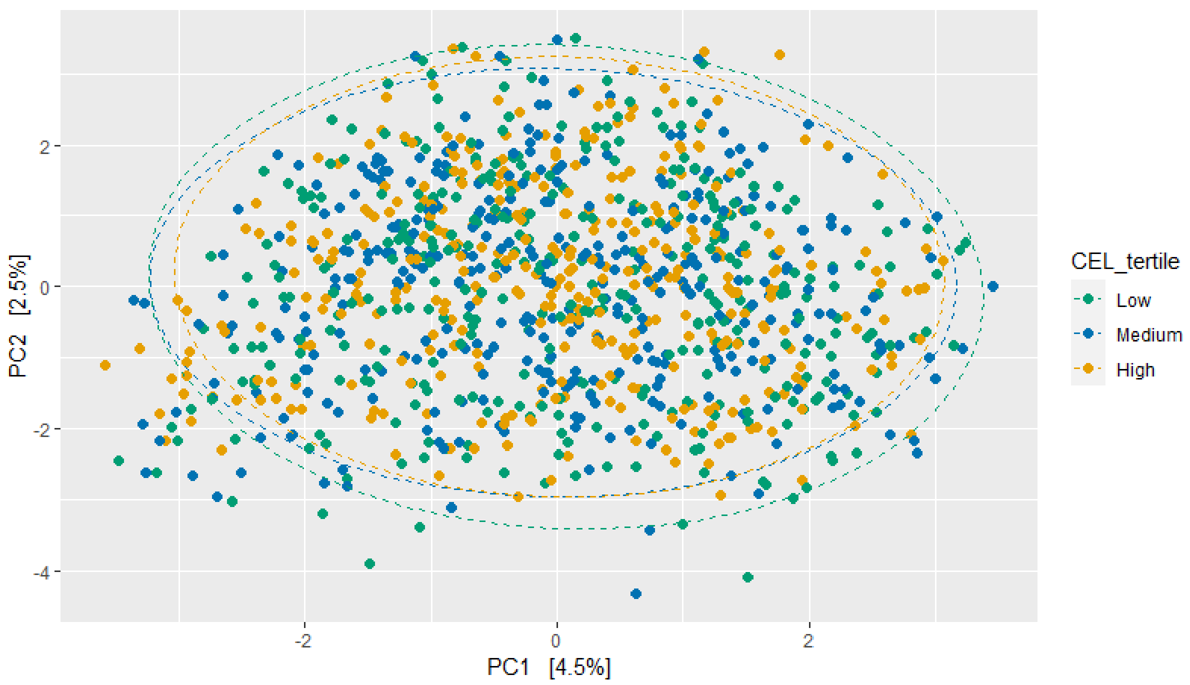

Two-dimensional ordination plot of microbiota distance by dAGE tertile groups based on the first two principal components of centered log-ratio transformed microbiota composition.

Figure S5 Ordination plot of microbiota beta dissimilarity among SAF tertile groups

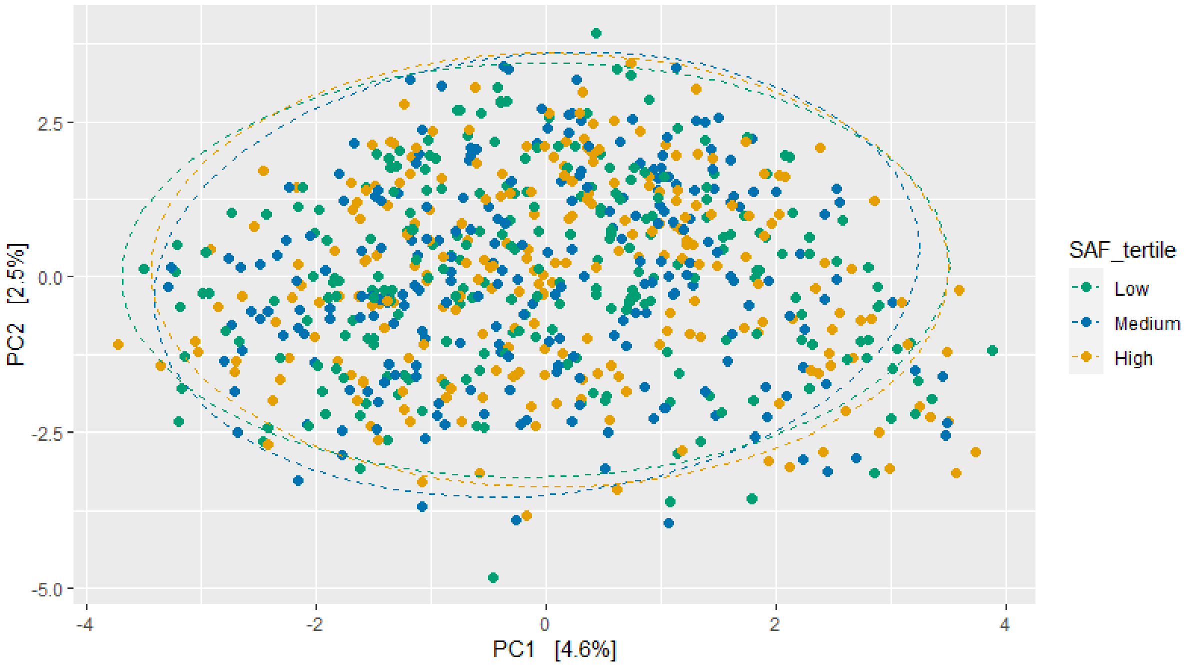

Two-dimensional ordination plot of microbiota distance by SAF tertile groups based on the first two principal components of centered log-ratio transformed microbiota composition.

Table S1 Associations between dietary AGEs intake and measures of alpha diversity in the total population

(A) Shannon Index

	Model 1		Model 2	
	β (95% CI)	p	β (95% CI)	p
CML	-0.02 (-0.04, 0.01)	0.3	-0.02 (-0.04, 0.01)	0.3
MGH1	-0.02 (-0.04, 0.01)	0.2	-0.02 (-0.05, 0.01)	0.1
CEL	-0.02 (-0.05, 0.01)	0.1	-0.02 (-0.04, 0.01)	0.2

(B) Inverse Simpson Index

	Model 1		Model 2	
	β (95% CI)	p	β (95% CI)	p
CML	-0.21 (-1.09, 0.67)	0.6	-0.20 (-1.09, 0.69)	0.7
MGH1	-0.27 (-1.15, 0.61)	0.6	-0.40 (-1.32, 0.52)	0.4
CEL	-0.26 (-1.13, 0.62)	0.6	-0.14 (-1.02, 0.74)	0.8

(C) Number of observed ASVs

	Model 1		Model 2	
	β (95% CI)	p	β (95% CI)	p
CML	-0.7 (-3.31, 1.9)	0.6	-0.78 (-3.36, 1.79)	0.6
MGH1	-1.24 (-3.85, 1.36)	0.3	-1.93 (-4.59, 0.73)	0.2
CEL	-2.03 (-4.63, 0.56)	0.1	-1.53 (-4.08, 1.02)	0.2

(A) The associations between dAGEs and Shannon Index; (B) The associations between dAGEs and Inverse Simpson Index; (3) The associations between dAGEs and number of observed ASVs.

Results are linear regression coefficients of z-score of dietary AGEs intake and the respective 95% confidence intervals obtained from 1052 participants, representing adjusted difference in the index associated with one SD higher of dAGEs. Indexes of alpha-diversity were derived from abundance of all observed ASVs.

Model 1: the association was adjusted for age, sex, season of stool production, number of total reads, and technical covariates (batches of DNA isolation and sequencing, and time in the mail).

Model 2: the association was adjusted for the use of PPI, and antibiotics, presence of diabetes, BMI, diet quality score, energy intake, alcohol intake, and smoking status in addition to model 1.

Table S2 Summary of PERMANOVA of beta dissimilarity by dAGEs tertile groups

(A)

Variables	R^2	p
Age	0.001	0.008
Sex	0.002	0.001
Season	0.004	0.001
Number of reads	0.006	0.001
Time in mail	0.001	0.02
DNA isolation batch	0.001	0.04
Sequencing batch	0.005	0.001
CML tertile groups	0.002	0.3

(B)

Variables	R^2	p
Age	0.001	0.004
Sex	0.002	0.001
Season	0.004	0.001
Number of reads	0.006	0.001
Time in mail	0.001	0.02
DNA isolation batch	0.001	0.03
Sequencing batch	0.005	0.001
PPI	0.003	0.001
Antibiotic use	0.003	0.001
Alcohol consumption	0.001	0.007
BMI	0.002	0.001
Diabetes	0.001	0.005
Dietary quality score	0.002	0.001
Energy intake	0.001	0.2
Smoking status	0.003	0.001
CML tertile groups	0.002	0.6

(C)

Variables	R^2	p
Age	0.001	0.007
Sex	0.002	0.001
Season	0.004	0.001
Number of reads	0.006	0.001
Time in mail	0.001	0.01
DNA isolation batch	0.001	0.04
Sequencing batch	0.005	0.001
MGH1 tertile groups	0.002	0.3

(D)

Variables	R^2	p
Age	0.001	0.004
Sex	0.002	0.001

Season	0.004	0.001
Number of reads	0.006	0.001
Time in mail	0.001	0.007
DNA isolation batch	0.001	0.05
Sequencing batch	0.005	0.001
PPI	0.003	0.001
Antibiotic use	0.003	0.001
Alcohol consumption	0.001	0.003
BMI	0.002	0.001
Diabetes	0.001	0.007
Dietary quality score	0.002	0.001
Energy intake	0.001	0.2
Smoking status	0.003	0.001
MGH1 tertile groups	0.002	0.3

(E)

Variables	R^2	p
Age	0.001	0.004
Sex	0.002	0.001
Season	0.004	0.001
Number of reads	0.006	0.001
Time in mail	0.001	0.02
DNA isolation batch	0.001	0.04
Sequencing batch	0.005	0.001
CEL tertile groups	0.002	0.1

(F)

Variables	R^2	p
Age	0.001	0.008
Sex	0.002	0.001
Season	0.004	0.001
Number of reads	0.006	0.001
Time in mail	0.001	0.01
DNA isolation batch	0.001	0.052
Sequencing batch	0.005	0.001
PPI	0.003	0.001
Antibiotic use	0.003	0.001
Alcohol consumption	0.001	0.004
BMI	0.002	0.001
Diabetes	0.001	0.005
Dietary quality score	0.002	0.001
Energy intake	0.001	0.2
Smoking status	0.003	0.001
CEL tertile groups	0.002	0.3

(A-B) Beta dissimilarity by CML tertile groups from model 1 and 2. (C-D) Beta dissimilarity by MGH1 tertile groups from model 1 and 2. (E-F)Beta dissimilarity by CEL tertile groups from model 1 and 2. PERMANOVA, permutational multivariate analysis of variance.

Results were obtained from PERMANOVA of 999 permutations in 1052 participants. Beta diversity was calculated as Aitchison distance, i. e., Euclidean distance based on centered-log ratio transformed abundance data of all ASVs. Variables were added into PERMANOVA sequentially (first to last).

Table S3-5 dAGEs-taxa associations in the total population

Provided as excel tables, with the genera presented in more than 30% of the participants highlighted.

N=1052 for Table S3 and 4, derived from model 1 and model 2, respectively N= 973 for Table S5 after further exclusion of participants with dAGEs intake outside of mean ± 4SD and those who had used antibiotics within 1-3 months before stool sample collection (results derived from model 2. 188 genera were analysed. Beta coefficient (95% CI) derived from linear regression models. It represents the adjusted difference (95% CI] of CLR transformed abundance of an individual ASV associated with one SD higher of dAGEs.

Model 1 was adjusted for age, sex, season of stool production, number of total reads, technical covariates (batches of DNA isolation and sequencing, and time in the mail).
Model 2 was additionally adjusted for the use of PPI, and antibiotics, presence of diabetes, BMI, diet quality score, energy intake, alcohol intake, and smoking status.

Table S6 dAGEs-pathway associations in the total population

Provided as excel tables, with the pathways presented in more than 30% of the participants highlighted.

N=1052, derived from model 2. Beta coefficient (95% CI) derived from linear regression models, representing the adjusted difference (95% CI] of CLR transformed abundance of an individual pathway associated with one SD higher of dAGEs.

Model 2 was adjusted for age, sex, season of stool production, number of total reads, technical covariates (batches of DNA isolation and sequencing, and time in the mail), the use of PPI, and antibiotics, presence of diabetes, BMI, diet quality score, energy intake, alcohol intake, and smoking status.

Table S7 Characteristics of participants included for stool microbiome and skin autofluorescence analyses and by tertile groups of skin autofluorescence

Characteristic	Overall	Sex-specific, age-adjusted SAF tertile		
		Low SAF	Medium SAF	High SAF
N	718	242	235	241
Age, y	63.4 ± 6.0	63.5 ± 5.75	63.1 ± 6.0	63.7 ± 6.2
Sex (female)	411 (57)	138 (57)	135 (57)	138 (57)
BMI	27.3 ± 4.6	26.85 ± 3.9	27.1 ± 4.0	27.8 ± 5.6
Smoking status				
never smoker	226 (32)	81 (34)	77 (33)	68 (28)
ex-smoker	370 (52)	133 (56)	127 (54)	110 (46)
current smoker	116 (16)	24 (10)	31 (13)	61 (26)
Physical activity	50.8 (24.6, 86.6)	52.5 (31.0, 91.5)	47.5 (20.9, 80.8)	50.2 (22.55, 86.8)
Alcohol consumption, g/day	8.6 (1.6, 8.6)	8.6 (1.6, 15.0)	8.6 (1.6, 8.6)	8.6 (1.6, 8.6)
Antibiotic usage				
No (n, %)	585 (81)	199 (82)	197 (84)	189 (78)
Within 1m before collection (n, %)	0 (0)	0 (0)	0 (0)	0 (0)
1m-3m before collection (n, %)	40 (6)	13 (5)	12 (5)	15 (6)
3m-1y before collection (n, %)	93 (13)	30 (12)	26 (11)	37 (15)
eGFR, mL/min/1.73 m^2	76.5 (68.0, 86.1)	76.7 (68.0, 86.2)	76.1 (68.55, 86.1)	76.4 (67.1, 86.0)
Diabetes	71 (10)	22 (9)	24 (10)	25 (10)
Use of PPI, n (%)	105 (15)	42 (17)	30 (12)	33 (14)
Season of sample production				
Spring (n, %)	211 (29)	85 (35)	65 (28)	61 (25)
Summer (n, %)	138 (19)	40 (17)	45 (19)	53 (22)
Autumn (n, %)	198 (28)	66 (27)	67 (29)	65 (27)
Winter (n, %)	171 (24)	51 (21)	58 (25)	62 (26)
Time in mail	1 (1, 1)	1 (1, 1)	1 (1, 1)	1 (1, 1)
Number of reads	26,339 (17,938, 33,449)	26,231 (18,476, 34,332)	26,417 (17,000, 33,014)	26,335 (17,638, 33,249)
SAF, A. U.	2.25 ± 0.43	1.85 ± 0.20	2.20 ± 0.17	2.70 ± 0.35

Microbial diversity				
Shannon Index	4.01 ± 0.43	4.03 ± 0.46	4.00 ± 0.41	4.00 ± 0.41
Inverse Simpson Index	33.2 ± 14.3	34.4 ± 14.4	32.6 ± 14.5	32.7 ± 14.1
Number of observed ASVs	158 ± 55	161 ± 55	158 ± 54	155 ± 55

BMI, body mass index; eGFR, estimated glomerular filtration rate; PPI, proton pump inhibitors; SAF, skin autofluorescence.

N=718. Values are shown for non-imputed data as counts (valid percentages) and means ± standard deviations, or as median (interquartile range) in case of a skewed distribution.

Table S8 The association between measures of alpha diversity and SAF

	Model 1		***Model 2***	
	β (95% CI)	p	β (95% CI)	p
Shannon Index	-0.06 (-0.13, 0.01)	0.1	-0.03 (-0.1, 0.04)	0.4
Inverse Simpson Index	-0.002 (-0.004, 0.0001)	0.06	-0.002 (-0.004, 0.0004)	0.1
Number of ASVs	-0.0005 (-0.001, 0.0002)	0.2	-0.0002 (-0.0009, 0.0005)	0.6

Coefficients and the respective 95% confidence intervals of the alpha-diversity indexes (Beta (95% CI)) were derived from linear regression analysis in 718 participants, representing adjusted difference in SAF associated with one-unit higher of the alpha-diversity index. Indexes of alpha-diversity were derived from abundance of all ASVs.

Model 1: the association was adjusted for age, sex, time in mail, DNA isolation and sequence batch, season of stool production, and number of reads.
Model 2: the association was adjusted for use of PPI and antibiotics, smoking status, diabetes status, eGFR, alcohol consumption, and BMI in addition to model 1.

Table S9-10 The associations between abundance of taxonomical units and SAF in the total population

Provided as excel tables, with the genera presented in more than 30% of the participants highlighted.

Coefficients and the respective 95% confidence intervals (Beta (95% CI)) were derived from linear regression model 1 and 2 of 718 participants. It is the adjusted difference in SAF associated with one unit higher of abundance of an individual ASV after CLR transformation.
Table S9: Model 1 was adjusted for age, sex, time in mail, DNA isolation and sequence batch, season of stool production, number of reads.
Table S10: Model 2 was further adjusted for the use of PPI and antibiotics, smoking status, diabetes status, eGFR, alcohol consumption, and BMI.

Table S11 Summary of PERMANOVA of beta dissimilarity by SAF tertile groups

(A)

Variables	R^2	p
Age	0.002	0.01
Sex	0.002	0.002
Season	0.006	0.001
Number of reads	0.006	0.001
Time in mail	0.002	0.006
DNA isolation batch	0.002	0.001
Sequencing batch	0.002	0.002
SAF tertile groups	0.003	0.2

(B)

Variables	R^2	p
Age	0.002	0.008
Sex	0.002	0.001
Season	0.006	0.001
Number of reads	0.006	0.001
Time in mail	0.002	0.006
DNA isolation batch	0.002	0.001
Sequencing batch	0.002	0.001
PPI	0.003	0.001
Antibiotic use	0.003	0.005
Alcohol consumption	0.002	0.04
BMI	0.003	0.001
Diabetes	0.002	0.008
eGFR	0.001	0.7
Smoking status	0.004	0.001
SAF tertile groups	0.003	0.6

(A-B) Beta dissimilarity by SAF tertile groups from model 1 and 2. PERMANOVA, permutational multivariate analysis of variance. Results were obtained from PERMANOVA of 999 permutations in 718 participants. Beta diversity was calculated as Aitchison distance, i. e., Euclidean distance based on centered-log ratio transformed abundance of all ASVs. SAF groups were categorized by sex-specific, age-adjusted SAF tertiles. Variables were added into PERMANOVA sequentially (first to last).

Table S12 The associations between microbial metabolic (MetaCyc) pathways and SAF in the total population

Provided as excel tables, with the pathways presented in more than 30% of the participants highlighted.

Coefficients and the respective 95% confidence intervals (Beta (95% CI)) were derived from linear regression model 2 of 718 participants. It is the adjusted difference in SAF associated with one unit higher of abundance of an individual pathway after CLR transformation.

Model 2 was further adjusted for the use of PPI and antibiotics, smoking status, diabetes status, eGFR, alcohol consumption, and BMI.
